# A pathogenic PSEN1 Trp165Cys mutation associated with early-onset Alzheimer’s disease

**DOI:** 10.1186/s12883-019-1419-y

**Published:** 2019-08-07

**Authors:** Vo Van Giau, Jung-Min Pyun, Jeewon Suh, Eva Bagyinszky, Seong Soo A. An, Sang Yun Kim

**Affiliations:** 1 Department of Bionano Technology & Gachon Bionano Research Institute, Gachon University, 1342 Sungnam-daero, Sujung-gu, Seongnam-si, Gyeonggi-do 461-701 South Korea; 2 Department of Neurology, Seoul National University College of Medicine & Neurocognitive Behavior Center, Seoul National University Bundang Hospital, 300 Gumidong, Bundang-gu, Seongnam-si, Gyeonggi-do 463-707 South Korea

**Keywords:** Pathogenic, PSEN1, Trp165Cys, Mutation, Alzheimer’s disease

## Abstract

**Background:**

Presenilin-1 (PSEN1) is one of the causative genes for early onset Alzheimer’s disease (EOAD). Recently, emerging studies reported several novel PSEN1 mutations among Asian. We describe a male with EOAD had a pathogenic PSEN1 mutation.

**Case presentation:**

A 53-year-old male presented with memory decline, followed by difficulty in finding ways. Patient had positive family history, since his mother and one of his brother was also affected with dementia. Brain magnetic resonance imaging (MRI) scan showed mild degree of atrophy of bilateral hippocampus and parietal lobe. ^18^F-Florbetaben-PET (FBB-PET) revealed increased amyloid deposition in bilateral frontal, parietal, temporal lobe and precuneus. Whole exome analysis revealed a heterozygous, probably pathogenic *PSEN1* (c.695G > T, p.W165C) mutation. Interestingly, Trp165Cys mutation is located in trans membrane (TM)-III region, which is conserved between PSEN1/PSEN2. In vitro studies revealed that *PSEN1* Trp165Cys could result in disturbances in amyloid metabolism. This prediction was confirmed by structure predictions and previous in vitro studies that the p.Trp165Cys could result in decreased Aβ42/Aβ40 ratios.

**Conclusion:**

We report a case of EOAD having a pathogenic *PSEN1* (Trp165Cys) confirmed with in silico and in vitro predictions.

## Background

Alzheimer’s disease (AD) (MIM #104300) is a neurodegenerative disease among elderlies, which is resulted by abnormal protein assembly inside the brain. Extracellular and intracellular amyloid beta (Aβ) and Tau protein, respectively, were associated as the main hallmarks of the disease. Early onset AD (EOAD) and late onset AD (LOAD) are the two main forms of the disease. Three genes were verified as causative factor for EOAD: amyloid precursor protein (*APP*) (MIM #104760) [1], presenilin 1 (*PSEN1*) (MIM #104311) [2], and presenilin 2 (*PSEN2*) (MIM #600759) [3]. Approximately 300 mutations of *PSEN1, PSEN2,* and *APP* in 635 affected individuals or families have been reported in the Dementia Mutation Database [4] (https://www.alzforum.org/mutations). Majority of mutations were observed in *PSEN1* [5–7] (*n* = 219, 76.6%) with over 230 mutations reported as pathogenic in the Alzforum database (https://www.alzforum.org/mutations/psen-1), as compared to *APP* (*n* = 51, 17.8%), and *PSEN2* (*n* = 16, 5.6%) [8–11].

PSEN1 protein contains nine transmembrane (TM) domains, connected with hydrophilic loop regions. As the member of γ- secretase complex, PSEN1 could function as a catalytic subunit of aspartyl protease, involved in the cleavage of C99 residue in APP protein into β-amyloid (Aβ) peptide. *PSEN1* mutations may impair the γ-secretase processing, resulting in altered of Aβ production. Gain-of-function mutations could increase the amyoid processing and the ratio Aβ42/Aβ40 [12, 13]. Loss- of- function mutations may reduce protective mechanisms, such as α-secretase cleavage [14]. In addition, accumulation of amyloid peptides may also associated with the reduced Aβ42 clearance [15] and neuronal loss [15–17]. In majority of patients, disease occurred at 40–50 years of age [18–21]. Several cases of young onset AD have been reported, where patients were less than 30–40 years of age [6, 7, 22–31]. Guerrio et al. (2010) designed an algorithm for variants in EOAD causative genes [32] on prediction of the pathogenic nature of novel mutations. Investigating patients carrying novel as well as previously known mutations along with the associated phenotypes will aid in classification of these variants and may eventually support genetic counseling [33]. In this study, we reported a pathogenic *PSEN1* W165C mutation as determined by genetic testing in a Korean patient with EOAD.

## Case presentation

**Fig. 1:**
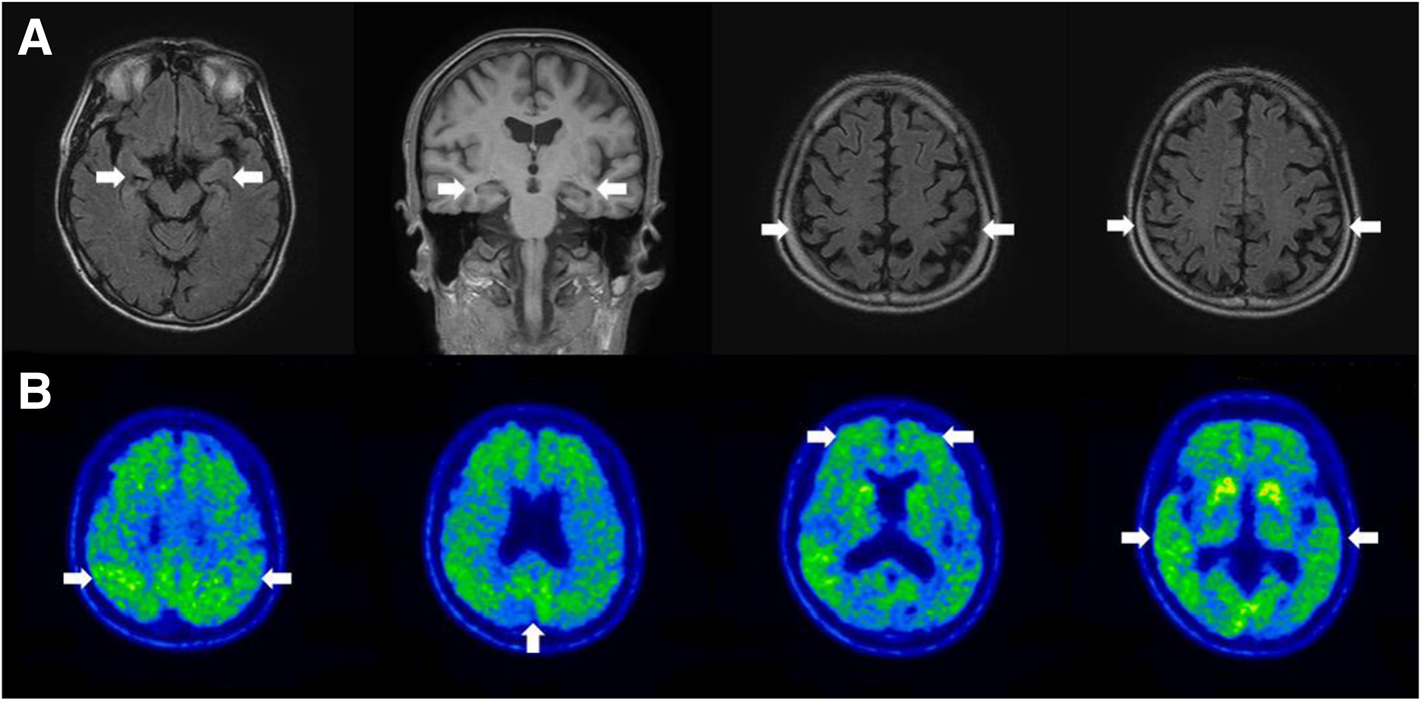
Brain functional and structural neuroimaging data of the proband at diagnosis. **a**. Axial FLAIR, coronal, and sagittal T1 images of brain MRI, arrows pointing at left-dominant bilateral temporal lobe atrophy. **b**. FDG-PET, arrows pointing at hypometabolism in left temporal cortex, right anterior temporal cortex and bilateral frontal cortex

A 53-year-old man with 13 years of education presented with progressive memory decline. At aged 50, he complained forgetfulness of meetings or details of story, and repeating the same questions. He had difficulty in orienting to date and in finding way to a new place. His past medical history revealed myocardial infarction with proper medical treatment. His Korean version of Mini-Mental Status Examination (K-MMSE) score was 21/30 and clinical dementia rating scale (CDR) score was 0.5 at three years after symptom onset. And follow-up K-MMSE score at six years after symptom onset was 19/30 and CDR score was 1. His brain MRI at three years after symptom onset revealed mild atrophy of bilateral hippocampus and parietal lobe (Fig. 1a**)**. FBB-PET at five years after symptom onset showed increased amyloid deposition in bilateral parietal, frontal, temporal lobe and precuneus (Fig. 1b**)**. The patient had an *APOE* ε2 /ε3 polymorphism.

**Fig. 2:**
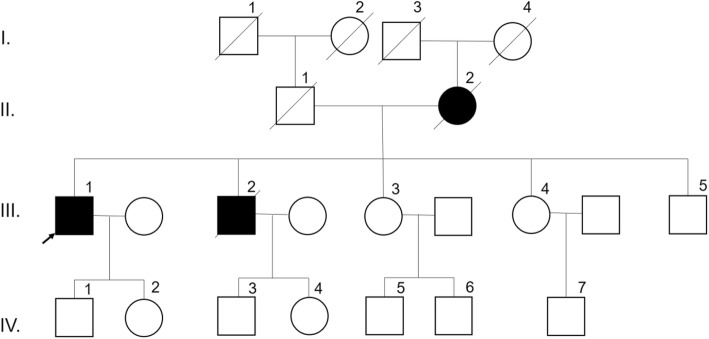
Family tree of patient with PSEN1 W165C mutation. White squares and circles mean asymptomatic family members, which were not diagnosed with disease. Family members which were crossed out, already died. Arrows show the proband patient

A 53-year-old man (III-1, Fig. 2) visited the Seoul National University Bundang Hospital with gradually impaired cognitive function over the previous years. The proband’s family history had a strong family history of dementia, and presented several family members affected by EOAD (Fig. 2). His mother (II-2) suffered from AD with onset in her fifties and deceased. The patient was one of the 4 siblings, comprising 2 brothers and 2 sisters. His first younger brother (III-2) was also diagnosed AD deceased in his forties, and had 2 children. His second younger brother (III-5) and his two younger sisters (III-3, III-4) displayed normal cognitive function. The health condition of the rest of his family members remained unknown, since all living family members and relatives declined to provide any additional information regarding their health.

## Genetic analysis of *PSEN1* and structural prediction of mutant PSEN 1 protein

### Methods

An in depth genetic screen was performed using a specifically expanded panel of 50 causative and risk factor genes for various neurodegenerative disorders [34]. Whole exome sequencing (WES) was performed in Novogene. Standard Sanger sequencing was carried out by BioNeer Inc. (Dajeon, Republic of Korea) [34]. Big Dye Terminator Cyclic sequencing was performed using the ABI 3730XL DNA Analyzer (Bioneer Inc., Dajeon, Republic of Korea). Sequencing data was analyzed using NCBI Blast (http://blast.ncbi.nlm.nih.gov/Blast.cgi) and the chromatograms were screened using the DNA BASER (http://www.dnabaser.com) tool. Possible novel mutations were checked in the Korean Reference Genome Database (KRGDB; http://nih.go.kr/menu.es?mid=a50303020300), which was obtained by whole genome sequencing of 622 healthy Korean individuals. The mutations were also screened against Broad Institute’s Genome Association Database (genome AD, http://gnomad.broadinstitute.org) and 1000 Genomes (http://www.1000genomes.org/) databases.

The possible pathogenic nature of missense variants was predicted using simple online tools, such as PolyPhen-2 (http://genetics.bwh.harvard.edu/pph2), Sorting Intolerant from Tolerant (SIFT; http://sift.jcvi.org/), and PROVEAN (http://provean.jcvi.org) algorithms. ExPasy analysis was also performed (https://www.expasy.org/) using different parameters, such as Kyte and Doolittle hydrophobicity index, bulkiness, and polarity. Mutant and normal prion protein structures were compared by 3D modelling. Protein structures were built using the Raptor X web server (http://raptorx.uchicago.edu/), while Discovery Studio 3.5 Visualizer (Accelrys, San Diego, USA) was used to display the 3D images [35].

## Results

**Fig. 3:**
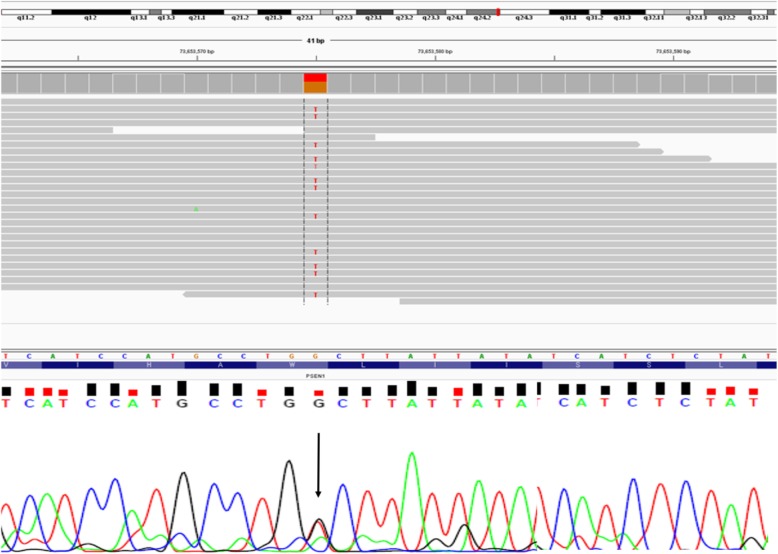
WES data of PSEN1 W165C mutation, verified by standard sequencing

A heterozygous G > T substitution (chr14; g.73653575: G > T) was discovered and confirmed to occur in the PSEN1 coding region using both WES and standard sequencing. This mutation caused the change from tryptophan to cysteine (c.495G > C; p.Trp165Cys) substitution, located at exon 6 of *PSEN1* gene, and in transmembrane (TM) helix domain-III of the PSEN1 protein (Fig. 3).

**Fig. 4:**
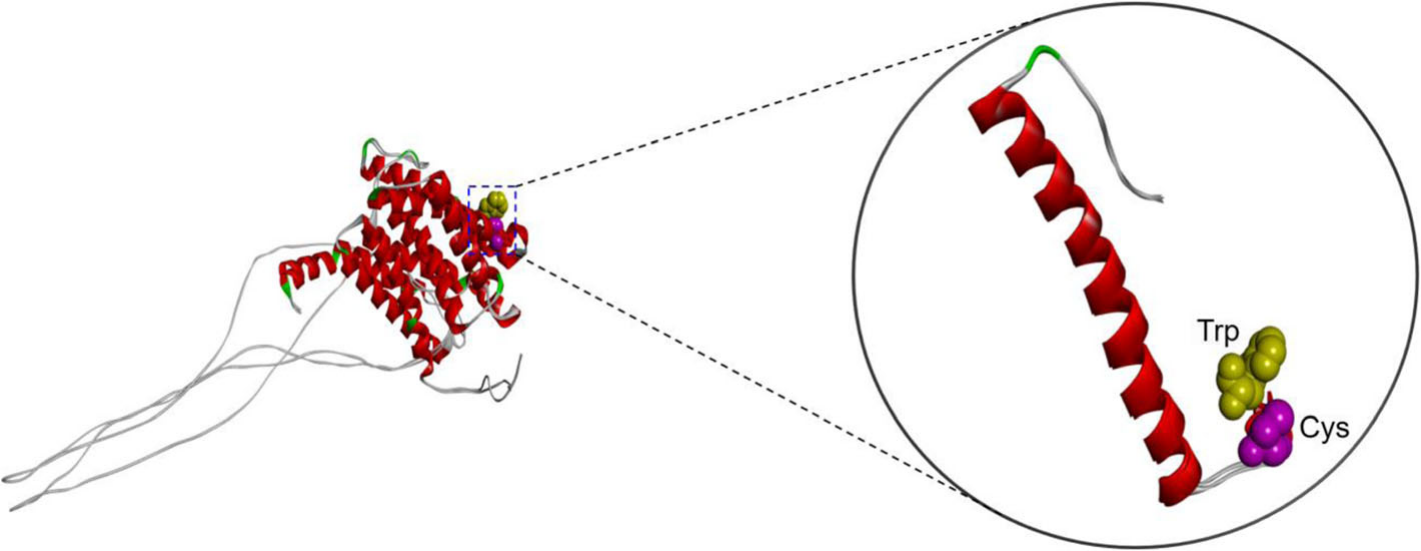
In silico structure predictions on PSEN1 W165C mutation. 3D modeling on PSEN1 Trp165Cys mutation, compared to the normal PSEN1. Alanine is labeled with blue while valine is labeled with yellow

**Fig. 5:**
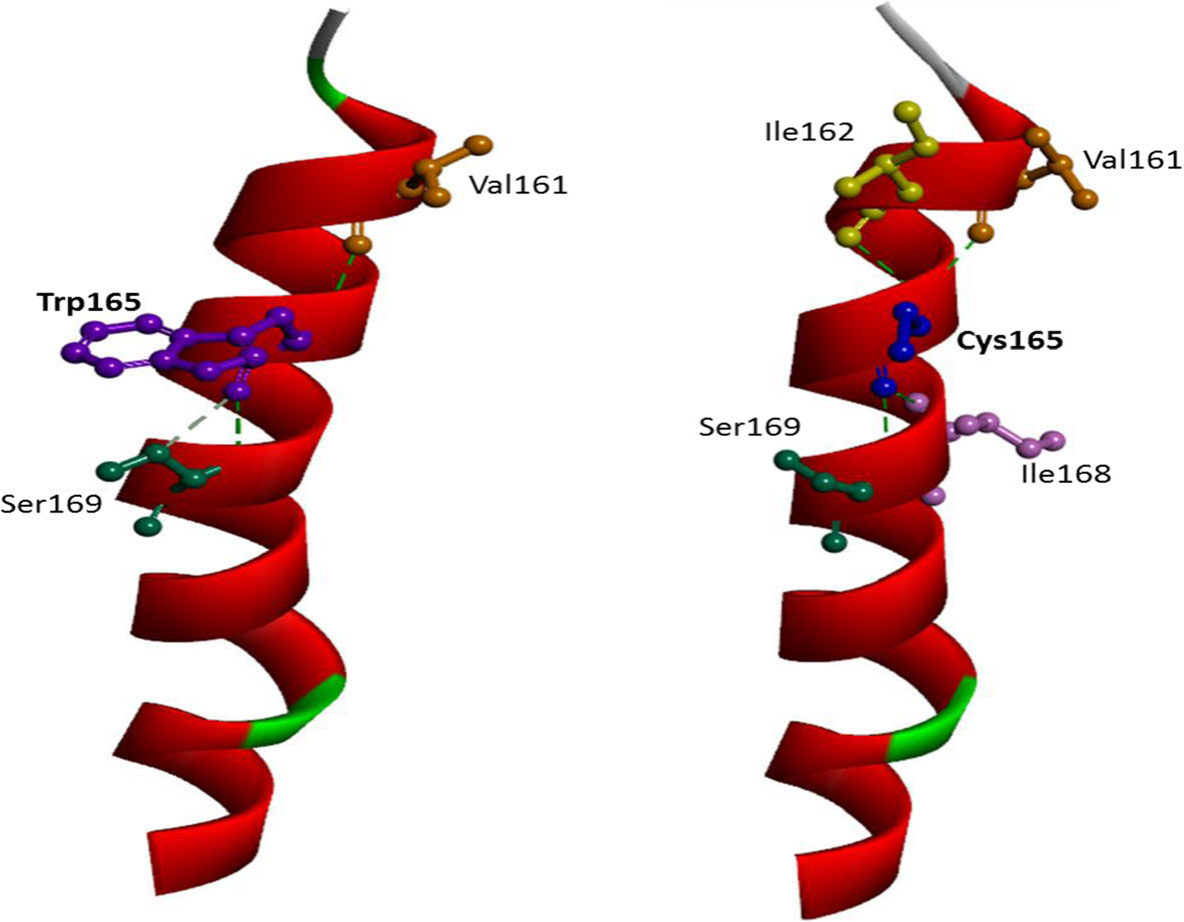
Putative intramolecular interactions in PSEN1 with Trp165 and PSEN1 with Cys165

The mutation is associated with EOAD patient with memory decline, followed by difficulty in finding ways and had a strong family history of AD; however, the specific mechanism is not functionally uncovered. The mutation was found in subject a Korean patient with EOAD and not observed in KRGDB, ExAC, and 1000genome control data sets. All in silico pathogenicity programs predicted the mutation to be deleterious. Figure 4 predicted that abnormal conformation inside the helix results in the mutated form due to an abnormal intra-or intermolecular disulfide bridge associated with the potential re-activities with metals or other compounds with thiol groups. Additionally, cysteine is not common recorded in the helix, further suggesting that this mutation might lead to abnormal conformation within the TM region. The intramolecular interactions may also change with the mutation: Trp165 has strong interaction with Ser169 (two hydrogen bonds), and forms another hydrogen bond with Val161. Cys165 changes the hydrogen bonds significantly. The interaction with Ser169 and Val 169 remained, but with Ser169, only one hydrogen bond was visible. Two new interactions could be seen with Ile162 and Ile168 (Fig. 5) In addition, the mutation is localized to trans membrane-III region conserved between PSEN1/PSEN2 and expected to affect Ab42 levels. This hypothesis was previously demonstrated that PSEN1 W165C led to increase Aβ42 and decreased Aβ40, resulting in elevated Aβ42/Aβ40 ratio in gaining loss of function in presenilin [36–39].

## Discussions

Since four different populations have been previously described the PSEN1 Trp165 from four familial AD cases (Table 1), the 165 codon seems to be a very vulnerable site. Initially, *PSEN1* Trp165Cys mutation was found in a French family, with a codon combination of TGG > TGC. Mutation was associated with strongly was positive family history, since several affected family members were identified in three generations. Age of onset ranged between 37 and 47 years in the relatives with disease. No details were available on clinical symptoms of affected patients [36]. Second case of Trp165Cys was discovered with alternative codon exchange of TGG > TGT in an Indian family. Affected patients developed disease in their 40s, and disease phenotypes were rapid progressive disease progression and cerebral/cerebellar atrophies [37]. Our case was associated with probable EOAD case in a male patient, and his family members presented AD in their 40s. It may be difficult to find out, whether there could be a common founder between the Indian and Korean families. Since the Korea and India may be geographically isolated from each other, we suggest that PSEN1 Trp165Cys occur independently in these two families.

**Table 1:** Clinical findings in the published at codon 165 of PSEN1

	Campion et al., 1999 [36] Wallon et al., 2012 [38]	Higuchi et al., 2000 [39]	Syama et al., 2018 [37]	This study
Country	France	Japan	India	Korea
Mutation	Trp165Cys	Trp165Gly	Trp165Cys	Trp165Cys
Codon change	TGG to TGC	TGG to GGG	TGG to TGT	TGG to TGT
Familiar	Yes 3 generation	Yes	Yes	Yes Two generations
Age at onset (year)	37–47	34–38	45	50
ApoE genotype	ε3/ε3	NA	NA	ε2/ε3
Clinical signs and symptoms	EOAD	EOAD	NA	Memory decline, followed by difficulty in finding ways and had a strong family history of dementia
Brain Imaging (MRI, CT)	NA	NA	MRI: indicated diffuse cerebral and cerebellar atrophy in one case.	18F-Florbetaben-PET (FBB-PET): increased amyloid deposition in bilateral frontal, parietal, temporal lobe and precuneus
Functional data	↑Aβ42 and ↓Aβ40 in vitro; elevated Aβ42/Aβ40 ratio	No functional data	↑Aβ42 and ↓Aβ40 production in vitro; elevated Aβ42/Aβ40 ratio	↑Aβ42 and ↓Aβ40 production in vitro; elevated Aβ42/Aβ40 ratio.

PSEN1 Trp165Cys is located in the TM-III region of PSEN1 protein. An exchange from native amino acid to Cys may increase the risk of an abnormal intramolecular disulfide bond formation with another Cys. These new S-S bonds may create novel inter- or intramolecular structures, involving in pathogenic mechanisms. The potential mechanism was displayed and revealed the Fig. 5. In addition, at the residue, another mutation to glycine (Gly, G) was previously documented in a Japanese family with young onset AD [39], suggesting that this residue may be critical for PSEN1 function. Interestingly, the mutation is located to TM-III region conserved between PSEN1/PSEN2 and expected to affect Ab42 levels. This prediction was previously demonstrated that PSEN1 Trp165Cys resulted in increased Aβ42 and decreased Aβ40, respectively. It could lead to elevated Aβ42/Aβ40 ratio in gaining loss of function in presenilin [36–39], verifying as a pathogenic mutation, involved in EOAD. This mutation was associated with rapid progression of disease, since the duration from the first clinical symptoms to the death ranged 4–10 years. Earlier onset of disease (37–47 years) was observed in a French family, but there was no information on the clinical symptoms in this family (Table 1).

**Fig. 6:**
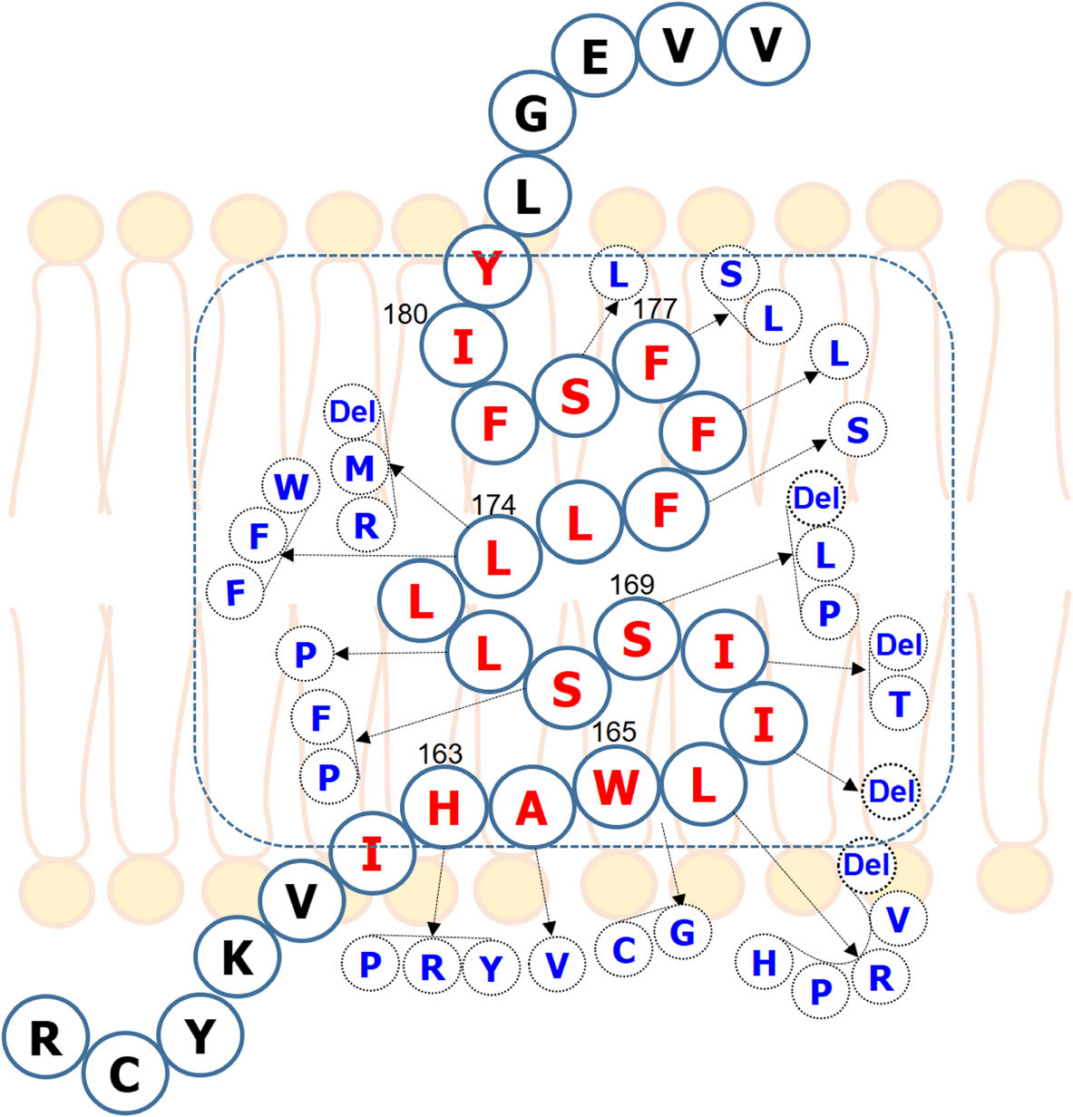
Mutations, discovered and located in the TM-III of PSEN1 protein

Several pathogenic *PSEN* genes are found to cluster within the predicted α-helical TMs. Among other TMs, TM3 has been identified as one of the critical site in PSEN1, where several familiar AD (FAD) mutations were found. Recently, more than twenty mutations associated with FAD have been reported in TM-III of PSEN1 [36, 40–50] (Fig. 6), and several of them were associated with EOAD (Table 2). Among these mutations, the PSEN1 Trp165Cys mutation is of particular interest because it may be associated with disease early onset. Moreover, the Trp165Cys mutation could cause increase in the Aβ42/Aβ total ratio [36–39], similarly to other FAD-associated PSEN1 H163R [51], H163Y [52], L166P [53], I167del [54], S170F [31] S170P [40], L174del [44], L173 W [36] and L174 M [45] mutations.

**Table 2:** Comparison of PSEN1 Trp165Cys with all mutations, located in membrane associated TM-III domain

Mutation	Clinical data	Age of Onset (Year)	Family History	Functional Studies	Reference
H163P	FLAIR, showing bilateral hippocampal, temporal lobe atrophy, and paraventricular hyperintensity. PET, revealing moderate-to-severe hypometabolism in the diffuse cortical area. Immunohistochemical stain revealed expression of amyloid beta in the senile plaque	34	No	↑Aβ42/Aβ40 ratio; ↑Aβ42; Aβ40.	[18] Kim et al., 2012
H163R	Data are limited, but neuropathology consistent with AD has been observed in at least one case.	42–47	Yes	↓Aβ42/Aβ total ratio in COS-1 cells; ↓Aβ42 and Aβ40 production in vitro. Involving γ-secretase- neurexin processing.	[51] Martin et al., 1995;
H163Y	Typical AD neuropathology (one case); decreased glucose metabolism in presymptomatic mutation carriers, especially in the thalamus. Widespread brain amyloid (PiB-PET) and shrunken hippocampi.	47	Yes	↓CSF Aβ42 and Aβ38 levels. ↑Aβ42/Aβ total ratio when expressed in COS-1 cells, and ↑Aβ42 production	[52] Clark et al., 1995
A164V	MRI revealed atrophy in brain involved anterior temporal lobe, and the hippocampus.	45–50	Yes	Possibly damaging via in silico.	[19] Roeber et al., 2015
W165C (G > C)	Not available	37–47	Yes	↑Aβ42 and ↓Aβ40; elevated Aβ42/Aβ40 ratio	[36] Campion et al., 1999
W165C (G > T)	EOAD, a severe form of atrophies and rapid deterioration in cerebral and cerebellar	45	Yes	↓Aβ42 and ↓Aβ40; elevated Aβ42/Aβ40 ratio	[37] Syama et al., 2018
W165G	Not available	34–38	Yes	Not available	[39] Higuchi et al., 2000
L166H	MRI: hippocampal atrophy and cortical atrophy. SPECT: bilateral hypometabolism in the parietal and frontal lobes.	30	Yes	Not available	[26] Pantieri et al., 2005
L166P	numerous Aβ-positive neuritic and cotton-wool plaques; abundant Aβ-positive amyloid cores in the cerebellar cortex.			↑Aβ42/Aβ ratio; ↓Aβ40, Aβ42, and AICD.↓cleavage of Notch and N-cadherin.	[53] Moehlmann et al., 2002
L166R	MRI revealed cortical atrophy; PET revealed parietal hypoperfusion.	32–34	Yes	Not available	[27] Ezquerra et al., 2000
L166 V	SPECT indicated temporoparietal hypoperfusion. Advanced plaques and tangles	42–50	Yes	Possibly damaging by in silico	[20] Sassi et al., 2014
L166del	MRI strongly revealed symmetrical cerebral atrophy,	40–46	Yes	Predicted possibly damaging in silico	[21] Knight et al., 2007
I167del	Symptom was progressive memory loss, behavior variants and spastic paraparesis.	38–46		Pathogenic by in silico	[54] Jiao et al., 2014
I168del	Not available		Yes	Pathogenic by in silico	[9] Janssen et al., 2003
I168T	Neuropathology consistent with AD.	86–94	Yes	Pathogenic by in silico	[20] Sassi et al., 2014
S169del (ΔS169, Ser169del, ΔS170)	MRI revealed cerebral atrophy involvement of the ventricles and widening of the sulci.	40	Yes	Not available	[28] Guo et al., 2010
S169 L	Aβ deposition in the cerebellum and white matter	33–37	Yes	Not available	[29] Taddei et al., 1998
S169P	Numerous plaques and neurofibrillary tangles could be seen in brain of the case	35	Yes	Not available	[30] Ezquerra et al., 1999
S170F	Much Lewy bodies in the substantia nigra and had severe cerebellar pathology Also, abundant amyloid deposition and loss of Purkinje cells reported.	27	Yes	↑Aβ42 and Aβ40, altering the ratio.	[31] Snider et al., 2005
S170P	MRI shown hypointensity, globus pallidus, and substantia nigra, as well as frontotemporal cortical atrophy. SPECT revealed severe nigrostriatal dopaminergic deficit bilaterally, and 18F-FDG PET hypometabolism in striatal and posterior cingulate.	25	No	Pathogenic by in silico	[40] Carecchio et al., 2017
L171P	Not available	36–40	Yes	Not available	[41] Ramirez-Dueñas et al., 1998
L173F (G > T)	Not available	50.5	Yes	↑Aβ42; ↑Aβ42:Aβ40 ratio	[42] Jin et al., 2012
L173F (G > C)	MRI revealed atrophy of the medial temporal lobe. SPECT indicated hypoperfusion of the posterior cingulate gyri and other cortical areas.	40–42	Yes	↑Aβ42 than cells ↑Aβ42:Aβ40 ratio	[43] Kasuga et al., 2009
L173 W	Not available	24–29	Yes	Not available	[36] Campion et al., 1999
L174del	MRI shown slight temporal lobe atrophy.	53	Yes	↑Aβ40, and ↓Aβ42 and Aβ42/Aβ40	[44] Tiedt et al., 2013
L174 M	Neuropathology consistent with AD and CAA associated	58	No	↓Aβ40 and ↑ Aβ42/Aβ40 ratio	[45] Tedde et al., 2003
L174R	EOAD	46–56	Yes	Not available	[46] Klünemann et al., 2004
F175S	Not available	NA	Yes	Not available	[47] Colacicco et al., 2002
F176 L	Much abundant amyloid plaques and neurofibrillary tangles in the cortex.	51	Yes	Not available	[48] Müller et al., 2013
F177 L	Not available	60	Not available	Not available	[49] Rogaeva et al., 2001
F177S	Not available	60	Not available	Not available	[49] Rogaeva et al., 2001
S178P	Not available	60	Not available	Not available	[49] Rogaeva et al., 2001

In our case, the PSEN1 p.Trp165Cys variant has been identified in a patient with early onset of age (50s years at diagnosis), suggesting that disease phenotype may be the result of amino acid substitution in this conservative residue. Furthermore, the amino acid position 165, located in the TM-III of PSEN1 indicated a significant phylogenetic conservation among vertebrates, and in homologous proteins such as PSEN2, suggesting that the position is of functional significance. Importantly, the patient’s mother and his brother were also affected by AD that is likely to involve autosomal dominant AD.

Guerrio et al. (2010) designed an algorithm on mutations on PSENs. which may be helpful in prediction on their pathogenic nature [32]. PSEN1 Trp165Cys may be a definitely pathogenic mutation. The Korean case of PSEN1 Trp165Cys may be associated with positive family history of disease, since the mother and one of the brother of patient was affected with AD. This is the third case of Trp165Cys, described all around the world, and EOAD was observed in all cases of AD. All of these findings suggested that Trp165 may be an important in PSEN1, since it bound two pathogenic mutations, Trp165Cys and Trp165Gly [36–38]. Functional studies, performed by Sun et al. (2016) revealed that mutation may impair the gamma secretase activity, resulting in elevated amyloid beta 42 production [55]. Our findings confirmed the significance of PSEN1 Trp165Cys in EOAD.

## Conclusions

We confirm that PSEN1 p.Trp165Cys may be commonly associated with EOAD. Our findings were consistent with the previously reported cases of this mutation, and supported the hypothesis that PSs contribute the identification of at risk relatives who may be potential candidates for clinical trials.

## Data Availability

Not applicable.

## Abbreviations

AD: Alzheimer’s disease
APP: Amyloid precursor protein
Aβ: β-amyloid
CDR: clinical dementia rating scale
EOAD: Early-onset Alzheimer’s disease
FDG-PET: Fludeoxyglucose- positron emission tomography
KRGDB: Korean Reference Genome Database
MMSE: Minimental state examination
MRI: Magnetic resonance imaging
PolyPhen2: Polymorphism phenotyping v2
PS1: Presenilin-1
PSEN1: Presenilin 1
PSEN2: Presenilin 2
SIFT: Sorting intolerant from tolerant
TM-III: Transmembrane segment III
WES: Whole exome sequencing
